# An Essential Role of INI1/hSNF5 Chromatin Remodeling Protein in HIV-1 Posttranscriptional Events and Gag/Gag-Pol Stability

**DOI:** 10.1128/JVI.00323-16

**Published:** 2016-10-14

**Authors:** Annalena La Porte, Jennifer Cano, Xuhong Wu, Doyel Mitra, Ganjam V. Kalpana

**Affiliations:** Departments of Genetics and Microbiology and Immunology, Albert Einstein College of Medicine, New York, New York; University of Illinois at Chicago

## Abstract

INI1/hSNF5/SMARCB1/BAF47 is an HIV-specific integrase (IN)-binding protein that influences HIV-1 transcription and particle production. INI1 binds to SAP18 (Sin3a-associated protein, 18 kDa), and both INI1 and SAP18 are incorporated into HIV-1 virions. To determine the significance of INI1 and the INI1-SAP18 interaction during HIV-1 replication, we isolated a panel of SAP18-interaction-defective (SID)-INI1 mutants using a yeast reverse two-hybrid screen. The SID-INI1 mutants, which retained the ability to bind to IN, cMYC, and INI1 but were impaired for binding to SAP18, were tested for their effects on HIV-1 particle production. SID-INI1 dramatically reduced the intracellular Gag/Gag-Pol protein levels and, in addition, decreased viral particle production. The SID-INI1-mediated effects were less dramatic in *trans* complementation assays using IN deletion mutant viruses with Vpr-reverse transcriptase (RT)-IN. SID-INI1 did not inhibit long-terminal-repeat (LTR)-mediated transcription, but it marginally decreased the steady-state *gag* RNA levels, suggesting a posttranscriptional effect. Pulse-chase analysis indicated that in SID-INI1-expressing cells, the pr55Gag levels decreased rapidly. RNA interference analysis indicated that small hairpin RNA (shRNA)-mediated knockdown of *INI1* reduced the intracellular Gag/Gag-Pol levels and further inhibited HIV-1 particle production. These results suggest that SID-INI1 mutants inhibit multiple stages of posttranscriptional events of HIV-1 replication, including intracellular Gag/Gag-Pol RNA and protein levels, which in turn inhibits assembly and particle production. Interfering INI1 leads to a decrease in particle production and Gag/Gag-Pol protein levels. Understanding the role of INI1 and SAP18 in HIV-1 replication is likely to provide novel insight into the stability of Gag/Gag-Pol, which may lead to the development of novel therapeutic strategies to inhibit HIV-1 late events.

**IMPORTANCE** Significant gaps exist in our current understanding of the mechanisms and host factors that influence HIV-1 posttranscriptional events, including *gag* RNA levels, Gag/Gag-Pol protein levels, assembly, and particle production. Our previous studies suggested that the IN-binding host factor INI1 plays a role in HIV-1 assembly. An ectopically expressed minimal IN-binding domain of INI1, S6, potently and selectively inhibited HIV-1 Gag/Gag-Pol trafficking and particle production. However, whether or not endogenous INI1 and its interacting partners, such as SAP18, are required for late events was unknown. Here, we report that endogenous INI1 and its interaction with SAP18 are necessary to maintain intracellular levels of Gag/Gag-Pol and for particle production. Interfering INI1 or the INI1-SAP18 interaction leads to the impairment of these processes, suggesting a novel strategy for inhibiting posttranscriptional events of HIV-1 replication.

## INTRODUCTION

Despite advances in the treatment of human immunodeficiency virus type 1 (HIV-1) infection, the AIDS pandemic remains unabated. The current FDA-approved antiretrovirals target entry, reverse transcription, integration, and virion morphogenesis but not transcriptional or posttranscriptional events that lead to particle production (1). During HIV-1 replication, transcription of the integrated provirus leads to the production of a single 9-kb transcript that is subsequently processed into singly or multiply spliced mRNAs. The unspliced viral RNA encodes pr55Gag (Gag) and pr160Gag-Pol precursor polyproteins (at a ratio of ∼20:1), which traffic through the cytoplasm to the plasma membrane for assembly and budding. A wealth of knowledge exists on the role of Gag and Gag-binding host proteins during assembly and budding (2–5). However, little is known about the role of Gag-Pol or the effects of Pol-binding proteins on assembly.

Genetic studies have demonstrated that mutations in the Pol region of Gag-Pol, comprising protease (PR), reverse transcriptase (RT), and integrase (IN), can lead to defects in particle morphology, virion release, uncoating, reverse transcription, or nuclear localization of the preintegration complex (6–13). The mechanism by which the Pol region within Gag-Pol influences these events is poorly understood. It is well established that Gag alone is sufficient for assembly and particle production. However, when Gag-Pol is present, mutations of IN have been shown to lead to defects in assembly and particle morphogenesis (14, 15). How IN or Pol mutations could influence assembly has not been elucidated. There are several hypotheses, one of which is that mutations in IN or Pol interfere with Gag and Gag-Pol oligomerization, thereby disrupting the assembly process (11, 13). Another hypothesis is that defects in IN may lead to premature protease action within the cells, and it has been shown that the inhibition of protease catalytic activity overcomes the assembly defects mediated by at least some of the IN mutants (12). A third hypothesis is that IN interacts with cellular proteins that are important for assembly. In this case, mutations within the IN region that disrupt interactions of Gag/Gag-Pol with cellular proteins would lead to assembly defects. In support of this hypothesis, we have previously demonstrated that *trans* dominant-negative mutants of the HIV-1 IN-interacting protein 1 (INI1)/hSNF5 that bind to the IN portion of Gag-Pol inhibit assembly in an IN-dependent manner (16, 17). Furthermore, INI1 interaction-defective mutants of HIV-1 IN (IID-IN) lead to defects in particle morphogenesis and infectivity (15). Additional studies have also shown that drugs (LEDGINs) that disrupt the interaction between IN and LEDGF, an essential cellular cofactor for IN, as well as allosteric inhibitors of IN (ALLINIs), impair particle morphogenesis and the infectivity of the virions (18, 19). It was subsequently demonstrated that ALLINIs increase the multimerization of IN (18, 20). These observations corroborate the hypothesis that Pol-binding host proteins or events that interfere with Pol function may influence the assembly process.

INI1/hSNF5, an HIV-1 IN-binding protein, is a tumor suppressor and a component of the chromatin remodeling, ATP-dependent SWI/SNF complex involved in transcriptional regulation (21). INI1/hSNF5 has multiple roles during HIV-1 replication. It has been shown to influence integration *in vitro*, long-terminal-repeat (LTR) transcription *in vivo*, and particle production in producer cells (21–29). The expression of an INI1 mutant comprised of the minimal IN-binding domain of INI1 (IBD; amino acids [aa] 183 to 294, also termed S6), potently and *trans* dominantly inhibits HIV-1 particle production without significantly affecting intracellular Gag/Gag-Pol levels (16). The inhibitory function of S6 {and a smaller fragment, S6 repeat 1 [S6(Rpt1)], aa 183 to 265} is correlated with the unmasking of a nuclear export sequence (NES) and its ectopic localization in the cytoplasm (16, 30, 31). INI1 selectively binds to HIV-1 but not other lentiviral INs, and it is incorporated into HIV-1 but not other lentiviral virions (17). The inhibition mediated by S6(Rpt1) is specific to HIV-1 (17). Together, these results indicate that an ectopically localized *trans* dominant-negative mutant of INI1 selectively binds to IN within the context of Gag-Pol to inhibit assembly and particle production. However, the mechanism by which S6, the INI1 mutant, inhibits HIV-1 assembly is not known, and whether endogenous INI1 is required for assembly remains unanswered.

To understand the role of INI1 in HIV-1 replication, it is important to know its cellular function. However, the function of INI1 within the SWI/SNF complex is not completely understood. It is thought to act as an adaptor protein that bridges the interaction of SWI/SNF components with sequence-specific transcription factors, thus recruiting the complex to the site of transcription (32). Because of INI1's ability to mediate multiple protein-protein interactions, we hypothesized that INI1-binding cellular proteins influence HIV-1 assembly and that identifying these cellular proteins would provide insight into the underlying mechanism. In an attempt to identify cellular proteins that bind to INI1 to understand its role in assembly, we previously screened a yeast two-hybrid library and identified SAP18 (Sin3a-associated protein, 18 kDa) as an interacting protein for INI1 (33). We demonstrated that SAP18 binds to INI1 and HIV-1 IN and that it is incorporated into HIV-1 (but not simian immunodeficiency virus [SIV] or human T-cell lymphotropic virus type 1 [HTLV-1]) virions (33). The role of SAP18 and its interaction with INI1 during assembly remains to be determined.

In this report, we have isolated and characterized a panel of SAP18-interaction-defective mutants of INI1 (SID-INI1) to determine whether the INI1-SAP18 interaction is required for HIV-1 particle production. The SID-INI1 mutants were isolated using a reverse yeast two-hybrid system. These SID-INI1 mutants are full-length INI1 proteins harboring substitutions that impair their interaction with SAP18. We characterized the effect of an SID-INI1 mutant that retained its ability to bind to IN and other INI1-binding proteins, such as cMYC, but was impaired for binding to SAP18. We found that SID-INI1 mutants exerted profound effects on HIV-1 particle production and impaired Gag levels and stability. Our analysis also indicated that the INI1 mutants did not affect LTR-mediated transcription but did affect Gag protein stability, suggesting that their effect is mediated at a posttranscriptional/translational level. Furthermore, in this report, we employed small hairpin RNA (shRNA)-mediated knockdown of endogenous INI1 in 293T cells. We found that full-length INI1 is required to maintain Gag levels, as well as for particle production. These studies suggest a role for INI1 in posttranscriptional events that affect Gag levels and particle production.

## MATERIALS AND METHODS

### Reverse yeast two-hybrid analysis and glutathione *S*-transferase (GST) pulldown assays.

The reverse two-hybrid assay was carried out essentially as described previously (34). In brief, a plasmid expressing a LexA-INI1 fusion protein (pSH2-INI1) was subjected to random mutagenesis to generate a random mutagenesis library using low-fidelity *Taq* polymerase. The plasmid library was screened against a fusion protein comprising the GAL4 activation domain fused to SAP18 (GAL4AC-SAP18) that was expressed from pGADNot-SAP18 in yeast. The X-Gal (5-bromo-4-chloro-3-indolyl-β-d-galactopyranoside) assay was used to identify INI1 mutants that resulted in white colonies due to INI1-SAP18 interaction defects. Interaction-defective clones were subjected to quality control by retesting to confirm their interaction defect and by subjecting protein isolated from white colonies to Western blot analysis to confirm stable expression of the interacting proteins. Plasmids were sequenced to identify mutations, and the specificity of the interaction defect was tested by their ability to interact with fusion proteins consisting of GAL4AC fused to cMYC, HIV-1 IN, and INI1. The strengths of interaction of the wild-type and the mutant INI1 with various INI1-interacting proteins were determined by the time of appearance of the blue color in the assay. The experiment was conducted at least three independent times to derive the results. Times of appearance within 0.5 h, 1 h, 2 h, and 3 h were assigned strengths of interaction of strong, moderately strong, weak, and very weak, respectively. The strongest interaction was between wild-type INI1 and HIV-1 IN.

The GST pulldown assay was performed essentially as described elsewhere, by expressing SID-INI1 mutants as 6His-tagged proteins in bacteria and testing for their ability to interact with GST-SAP18 immobilized onto glutathione agarose beasds (G-beads) (33).

### Viral DNA transfection, viral particle production, and p24/p27 ELISA.

The production and analysis of the HIV-1 three-plasmid-based vector (3pV) and HIV-1_NL4-3_ full-length molecular clone were carried out as described elsewhere (30). The 3pV plasmid-based-vector system is a kind gift of Trono and consists of the following three plasmids, which were cotransfected in a ratio of 1:2:1, respectively: (i) pMDG, expressing vesicular stomatitis virus envelope glycoprotein (VSVg) for pseudotyping; (ii) pHR'CMV-GFP, a transducing vector expressing green fluorescent protein (GFP); and (iii) pCMVΔR8.2, expressing Gag-Pol under the cytomegalovirus (CMV) promoter. During the transfections, a 15-fold excess of the plasmid encoding the INI1 mutant to the plasmid expressing Gag-Pol was used. The effects of INI1 mutants were monitored when the transfection efficiency was 90% or greater. The viral supernatants were collected 24 h after the medium was switched. SIV-based vectors (a kind gift of A. Nienhuis, St. Jude's) were prepared using pCL20cSLFRMSCV-GFP, pCAG-SIVgprre, pCAG4-RTR-SIV, and pMDM-VSVg in a 5:3:1:1 ratio (35). The protease mutants of HIV-1 were a kind gift of E. Freed (36). The HIV-1 vectors used for the IN *trans* complementation assay, SG3-IN and Vpr-RT-IN, were as reported previously (16, 37). The HIV-1 viral capsid levels in the medium were determined by measuring p24 levels using either an enzyme-linked immunosorbent assay (ELISA) (catalog number 5421; Advanced Bioscience Laboratories) or the AlphaLISA kit (product number AL207F; PerkinElmer). The SIV capsid levels were measured by determining p27 CA levels using an ELISA kit (catalog number 5436; Advanced Bioscience Laboratories).

### Methylene blue uptake assay to determine cell viability.

293T cells were transfected with pNL4-3 and INI1 or SID-INI1 or shRNA constructs as described above, and after transfection, the cells were washed and then treated with 0.5% methylene blue (formulated in a 1:1 ratio of ethanol-water [vol/vol]) and incubated at room temperature for 1 h. After treatment, the cells were washed and dried. To each well, 1 ml of 1% SDS was added to solubilize cell membranes. The plates were incubated with agitation on a rotator at room temperature for 1 h. The resulting cell lysate was collected, and the methylene blue was measured by reading the optical density at 630 nm (OD_630_) of the sample from each well.

### Dual-luciferase assay to determine the effect on LTR transcription.

293T cells were transfected with 2 μg pCGN-INI1 or pCGN-SID-INI1, 200 ng of pLTR-Luc, 10 ng of pCMV-Renilla, 200 ng of GFP, and 50 ng of pcDNA-tat and/or pcDNA to a total of 3 μg in 12-well plates. Cells were collected 24 h posttransfection and lysed in 50 μl passive lysis buffer (PLB), and 10-μl amounts were added in triplicate to a white-walled 96-well plate. Luciferase was measured using a Victor2 multiplate reader (Perkin-Elmer). Luminometer readings were set to a sequence of 2-s premeasurement delay, addition of LARII reagent, 10-s measurement, 2-s premeasurement delay, addition of Stop & Glo, and 10-s measurement. Values were normalized to total protein concentration.

### Reverse transcription-qPCR to determine the levels of gag RNA.

Total RNA (2 μg) samples extracted from cells expressing HIV-1 and INI1 and SID-INI1 using an RNeasy kit (catalog number 74104; Qiagen) were treated with DNase I (Applied Biosystems) and subjected to reverse transcription. An amount of 6.4 ng of viral cDNA was subjected to quantitative PCR (qPCR) using 2× Sybr green master mix (Fermentas) or TaqMan master mix (catalog number 4444556; Thermo Fisher Scientific) and the following late reverse transcription primers that amplify *gag*: forward, 5′-TGTGTGCCCGTCTGTTGTGT-3′, and reverse, 5′-GAGTCCTGCGTCGAGAGAGC-3′. qPCR was performed using a ABI 7700 real-time PCR machine and analyzed using SDS2.1 software.

### Pulse-chase (^35^S) analysis of viral proteins.

293T cells were transfected with 2 μg of HIV-1 and 30 μg of pCGN-INI1 or pCGN-SID-INI1-B by using the calcium chloride precipitation method. The medium was changed after 16 h, and the culture incubated for an additional 2 h and labeled with [^35^S]Met-Cys (catalog number NEG072; Perkin-Elmer). After labeling, the cells were washed in Dulbecco modified Eagle medium without methionine or cysteine [DMEM(−Cys/−Met)] (catalog number D0422; Sigma), resuspended in 5 ml of DMEM(−Cys/−Met), and incubated at 37°C for 30 min to starve the cells. The medium was removed and replaced with 3 ml of DMEM(−Cys/−Met) plus 2% fetal bovine serum (FBS). [^35^S]Met-Cys was added to each plate at a concentration of 250 μCi/ml in 3 ml and incubated for 1 h at 37°C. The medium was removed, the cells were washed once with DMEM, and 10 ml of chase medium (DMEM plus 10% FBS, 5 mM methionine, and 5 mM cysteine) was added to each plate. The supernatants and cell pellets were collected at 0, 2, 4, and 6 h postchase.

### Isolation of ^35^S-labeled viral particles and intracellular ^35^S-labeled viral proteins.

To harvest virus, supernatants were clarified by low-speed centrifugation, filtered through a 0.45-μm cellulose acetate syringe filter (catalog number 431220; Corning), and ultracentrifuged in a polyallomer tube (catalog number 331372; Beckman) at 35,000 rpm for 45 min at 4°C. The supernatant was removed, and the virus pellet was resuspended in 1 ml of phosphate-buffered saline (PBS). The sample was incubated at room temperature for 15 min and brought up to 4 ml with PBS. The virus pellet was then subjected to a step gradient centrifugation using a 20% sucrose cushion at 35,000 rpm for 2 h at 4°C. The supernatant was removed and the pellet resuspended in 1 ml of lysis buffer (0.1 mM EDTA, 150 mM NaCl, 5 mM MgCl_2_, 5 mM CaCl2, 1 mM dithiothreitol [DTT], 20 mM HEPES-KOH, pH 7.9, 1% Triton X-100, protease inhibitor tablet [1 per 10 ml]). Meanwhile, the intracellular proteins were collected by washing cells once with PBS and resuspending them in 1 ml of lysis buffer.

### Immunoprecipitation of intracellular HIV Gag.

After the addition of lysis buffer, cell lysates and viral supernatants were incubated at 4°C for 1 h with rotation. Samples were spun briefly to pellet debris, the supernatant transferred to a new tube, and the pellet discarded. The supernatant was precleared with 40 μl of protein G Dynabeads (catalog number 100-03D; Invitrogen) for 2 h at 4°C with rotation. The supernatants were briefly centrifuged to pellet the beads, and the supernatant was then transferred to a new tube and the beads discarded. Two microliters of anti-p24 antibody was added to each sample. The samples were incubated overnight at 4°C with rotation. The next day, 40 μl of PBS-washed protein G Dynabeads were added to the samples, which were then incubated for an additional 3 h. The beads were washed 5 times with 500 μl to 1 ml of wash buffer (20 mM HEPES-KOH, pH 7.9, 150 mM NaCl, 0.1 mM EDTA, 1 mM DTT, 1% Triton X-100, protease inhibitor tablet [1 per 10 ml]), resuspended in 20 μl of PBS followed by 20 μl of 5× SDS dye, and loaded onto a 15% SDS–polyacrylamide gel. The gel was fixed with 40% methanol, 10% acetic acid in water for 1 h, rinsed for 10 min in water, and treated with 25 ml of 1 M salicylic acid (in water) containing 2% glycerol for 1 h. The gel was dried for 1.5 h using a gel dryer set to 80°C. The dried gel was exposed for 2 days to X-ray film.

### Confocal microscopy.

Cells were transfected using the CaCl_2_ precipitation method at 10 to 30% confluence in 2- or 4-well chamber slides (catalog number 177399; Nunc) using 0.063 μg pNL4-3 and 0.94 μg plasmids, pCGN-INI1, or pCGN-INI1-B per chamber. The medium was changed 16 h after the addition of DNA, and cells were fixed and stained 24 h after the medium was changed. The cells were fixed using Eddy fix (38) (3.7% paraformaldehyde, 0.1% glutaraldehyde, 0.15 mg/ml saponin in PBS) for 15 min at room temperature, washed three times with PBS, and permeabilized with 0.5% Triton X-100 for 10 min at room temperature, followed by RNase treatment for 45 min at 37°C. Cells were then washed and blocked for 1 h with 2.5% bovine serum (catalog number A2153; Sigma) and incubated with anti-p24 antibody at 1:500 and anti-hemagglutinin (HA)-fluorescein isothiocyanate (FITC) antibody (catalog number H7411; Sigma) at 1:750 in 1.5% bovine serum albumin (BSA) for 3 h at 37°C, followed by three washes with PBS. Samples were then incubated with anti-phycoerythrin (PE)-conjugated secondary antibody or anti-FITC antibody, for control purposes, at 37°C for 1 h. Cells were subsequently washed three times with PBS. A coverslip (catalog number 125485E; Fisher) was mounted using mounting medium containing 4′,6-diamidino-2-phenylindole (DAPI) (catalog number H-1200; Vector Laboratories). Images were collected sequentially using a Leica sp2 or sp5 confocal microscope at ×63 magnification and 6.5× zoom, with a line average of 4. Z-stacks were taken as 0.5-μm slices with a line average of 4. Images were analyzed using ImageJ (and Photoshop) software and saved as TIFF files.

### Immunohistochemistry.

The INI1 antibodies that are available have been used successfully in immunohistochemical studies on tumor sections with antigen retrieval (39). However, these antibodies did not work well on cultured cells. Therefore, we developed a method to detect endogenous INI1 protein, along with p24, in shRNA-treated cells based on the established immunohistochemical methods. With the help of the Albert Einstein histopathology core, a method for the detection of endogenous INI1 was developed using anti-BAF47 antibody (catalog number 612110; BD Biosciences). After transfection with shRNAs and pNL4-3, the 293T cells were washed and fixed on the plate for 15 min with 4% formalin. The cells were collected, washed, and resuspended in 200 to 300 μl of liquefied HistoGel, and the mixture was placed into a plastic mold for 1 h at room temperature. The cell-containing gel blocks were then embedded in wax blocks, which were cut into slices and placed onto slides. This procedure is very similar to the one used for cells in suspension in preparation for microscopy. Immunohistochemistry was subsequently performed on the sections containing the cells, following antigen retrieval by heating in a steamer for 20 min with 1 mM EDTA, 10 mM Tris-Cl, pH 9, and cooling for 30 min at room temperature, followed by 2 min of washing with Tris-buffered saline (TBS). The slides were dried, and a circle was drawn around the samples using a PAP pen. The samples were then washed 3 times with TBS, followed by blocking for 1 h with 5% donkey serum, 2% BSA, 0.3 M glycine, 0.4% Triton X-100 in PBS. After blocking, the samples were incubated in a moist chamber with the primary antibodies anti-BAF47 antibody (catalog number 612110; BD Biosciences) and anti-p24 antibody (a gift from David Ott) overnight at 4°C or with IgG control in 5% BSA. The next day, the samples were washed well with TBS (4 times for 5 min each) and incubated with the secondary antibodies anti-goat-PE antibody (catalog number sc-3747), anti-rabbit-Alexa Fluor 594 antibody (catalog number A-21207; Life Technologies), and anti-mouse-Alexa Fluor 488 antibody (catalog number A-21202; Life Technologies), respectively, for 1 h at room temperature. Samples were washed 2 times for 5 min each and cleared in xylene for 3 min, and a coverslip was mounted using DAPI mounting medium. Image capture and analysis were carried out as described above.

## RESULTS

### Isolation of INI1 mutants defective for binding to SAP18.

**FIG 1:**
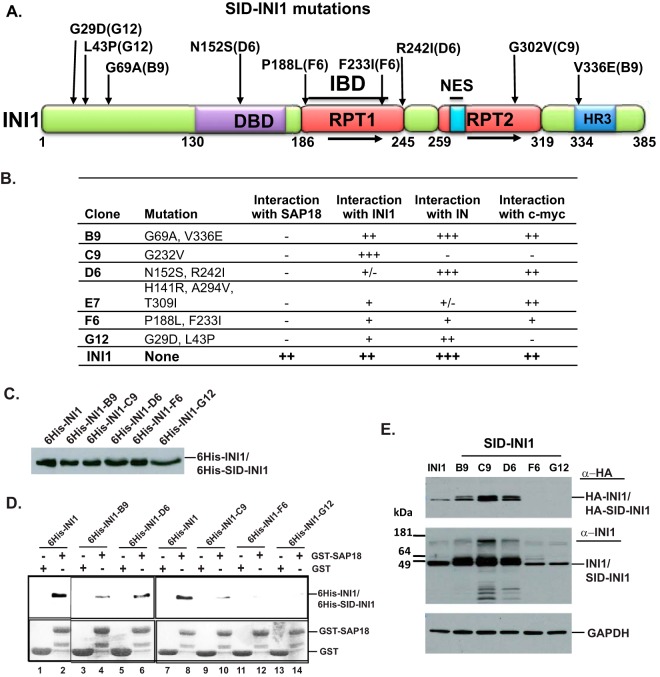
Isolation and characterization of SID-INI1 mutants. (A) Cartoon of INI1, illustrating the positions of mutations that result in defects in binding to SAP18. The designations of the clones bearing the mutations are in parentheses. RPT, repeat; HR3, homology region 3; NES, nuclear export sequence; IBD, minimal integrase binding domain; DBD, nonspecific DNA binding domain. (B) Table indicating the SID-INI1 mutations and their interaction with SAP18 and other INI1-interacting proteins. −, no interaction; +/−, very weak interaction; +, weak interaction; ++, moderately strong interaction; +++, strong interaction. (C and D) Immunoblot analysis with anti-6His antibody of 6His-INI1 and 6His-SID-INI1 mutants expressed in bacteria (C) and *in vitro* GST-pulldown assay showing the interactions between the 6His-INI1 or SID-INI1 mutant proteins and GST or GST-SAP18 (D). (D) Top, bound 6His-INI1 and 6His-SID-INI1 proteins; bottom, loading of GST and GST-SAP18 proteins. (E) Immunoblot analysis of the expression of HA-SID-INI1 mutants in 293T cells, using anti-HA-horseradish peroxidase (HRP), anti-BAF47 (INI1), and anti-GAPDH antibodies. While anti-HA antibody detects only the transfected HA-tagged proteins, anti-BAF47 (INI1) antibody detects both endogenous and transfected HA-tagged INI1 proteins. HA-SID-INI1 clones F6 and G12 are not expressed in 293T cells and, hence, are not detected by the anti-HA antibodies (top). The band that appears in the F6 and G12 lanes (middle) corresponds to the endogenous INI1 protein, detected by anti-BAF47 (INI1) antibody. Lanes 1 to 6 in panel D are taken from different areas of the same gel.

To determine the importance of INI1's interaction with SAP18, we isolated mutants of INI1 unable to bind to SAP18 (SID-INI1) by using a reverse yeast two-hybrid system (for methods, see reference 34). Briefly, a random mutation library of INI1 was generated using the low-fidelity *Taq* polymerase and the products cloned into a yeast two-hybrid system vector, pSH2-1, as fusion proteins fused to the LexA DNA-binding domain. The library was screened against SAP18 expressed as a fusion protein fused to the GAL4-activation domain, and interaction was detected by the activation of the *LacZ* reporter gene. Interaction between INI1 and SAP18 resulted in blue colonies in the yeast two-hybrid system, and a lack of interaction resulted in white colonies. The clones isolated from white colonies were subjected to a series of quality control experiments, including (i) isolation and restriction digestion of the plasmid to ensure the presence of the insert and lack of rearrangements, (ii) retesting for the interaction defect by retransformation of the plasmid into yeast, and (iii) testing for stable expression of proteins (34). Those clones that passed the quality control testing were sequenced, and the positions of the mutations in INI1 were identified (Fig. 1A and B). As another quality control and to determine the specificity of the interaction defect, the SID-INI1 clones were tested for their ability to bind to other INI1-interacting proteins, such as INI1 (for dimerization), HIV-1 IN, and cMYC, using a yeast two-hybrid system (Fig. 1B). Clones B9 (termed SID-INI1-B) and D6 (termed SID-INI1-D) were selectively defective for binding to SAP18 but retained their ability to bind to INI1, IN, and cMYC (Fig. 1B). Clone C9 exhibited a defect in its interactions with all INI1 binding partners except INI1 (Fig. 1B). Clone F7 exhibited defects in interactions with all INI1 interaction-defective mutants except cMYC (Fig. 1B). The remaining clones, F6 and G12, exhibited generally weak interactions with all partners tested (Fig. 1B).

To confirm the yeast two-hybrid results, we also carried out an *in vitro* GST pulldown assay using hexahistidine (6His)-tagged SID-INI1 mutants (except for E7, which carried three substitutions) and GST-SAP18, expressed in bacteria. First, the expression levels of the various mutants were analyzed using immunoblot analysis. Equal levels of proteins were used for *in vitro* binding (Fig. 1C). The binding reactions were subjected to immunoblot analysis using anti-6His antibody to detect the bound wild-type INI1 and mutant proteins. The results of these analyses indicated that, while the SID-INI1 mutants were completely defective for binding to SAP18 in a two-hybrid screen, they were partially or fully defective for binding to GST-SAP18 using the *in vitro* pulldown assay. Clones B9, D6, and C9 were partially defective, and clones F6 and G12 were fully defective (Fig. 1D).

### SID-INI1 mutants selectively inhibit HIV-1 particle production at two distinct stages.

**FIG 2:**
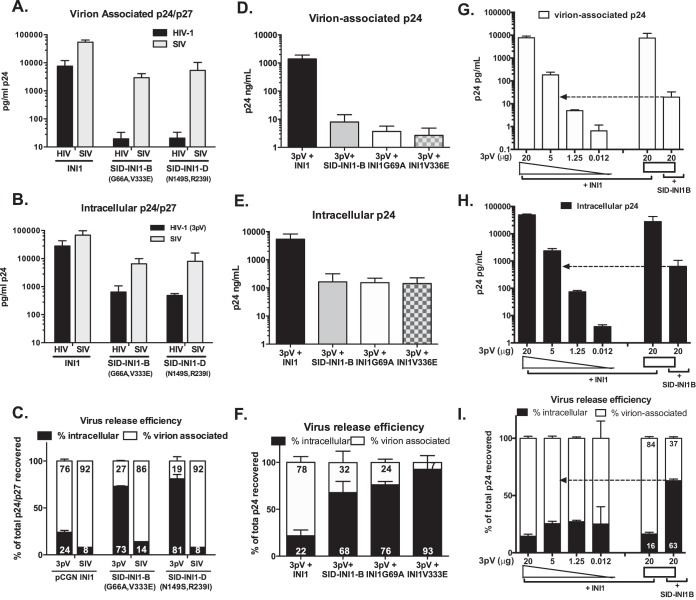
Effects of SID-INI1 mutants on particle production of HIV-1- and SIV-based vectors. (A and B) Quantitation of virion-associated (A) and intracellular (B) p24 (to measure HIV-1-based vectors) and p27 (to measure SIV-based vectors). (C) Virus particle production efficiency, measured by calculating percentages of virion-associated and intracellular fractions of p24/p27 compared to total p24. (D to F) Effects of HA-SID-INI1-B and of single mutations in HIV-1 3pV were measured using p24 ELISA, as described in the legends to panels A to C. (G to I) The effects of varying intracellular levels of Gag were determined by transfecting decreasing amounts of 3pV viral DNA to match the Gag levels in cells transfected with SID-INI1-B. Virion-associated and intracellular p24 and particle production efficiency were measured as described in the legends to panels A to C. Graphs represents mean values from 3 or more independent experiments ± standard errors of the means (SEM). The arrows point to the control sample with Gag levels comparable to that for the SID-INI1B-transfected cells.

To determine the effect of expressing SID-INI1 mutants on HIV-1 replication, we chose mutants B9 (bearing substitutions of A for G at position 69 and E for V at position 336 [G69A V336E]; referred to as SID-INI1-B) and D6 (N152S R242I; referred to as SID-INI1-D) for further study for the following reasons. These mutants were specifically defective in binding to SAP18 but retained the ability to bind to INI1, IN, and cMYC in the yeast two-hybrid system (Fig. 1B), and they were well expressed in 293T cells (Fig. 1E, top). In contrast, mutant C9 exhibited defective binding to IN, c-Myc, and SAP18 and, hence, was not specific to SAP18. Although mutants F6 and G12 appeared to be specifically defective for binding to SAP18, they were not well expressed in 293T cells (Fig. 1E, top). For these reasons, C9, F6, and G12 were not studied further. Of the two mutants B9 and D6, which exhibited very similar effects, we chose mutant B9 for further study, and we termed it SID-INI1-B. First, we examined the effects of HA-SID-INI1 mutants on the particle production of the three-plasmid-based HIV-1 vector (3pV) in 293T cells, where Gag-Pol is expressed under the control of a CMV promoter. We found that there was an ∼3-log (∼1,000-fold) decrease in the level of p24 in the culture supernatant and ∼1- to 2-log decreases (10- to 40-fold) in intracellular p24 when the HA-SID-INI1-B and -D mutants were expressed (Fig. 2A and B). We also calculated the particle production efficiency by determining the fractions of intracellular and culture supernatant-associated p24 and comparing these to the total p24 in a given sample (expressed as percentages of the total). The HA-SID-INI1 mutants reduced the intracellular Gag levels, as well as the particle production efficiency (Fig. 2A to C). While control cells expressing HA-INI1 released 76% of the total p24 into culture supernatants, cells expressing the HA-SID-INI1 mutants released 19 to 27% of the total p24 (Fig. 2C).

To rule out the possibility that the HA-SID-INI1-mediated inhibition was due to nonspecific effects on cellular functions, we tested the influence of these mutants on the replication of simian immunodeficiency virus (SIV)-based vectors. SIV IN does not interact with INI1 (human and macaque INI1 proteins are 100% conserved), and neither INI1 nor SAP18 is recruited into SIV virions (16). The HA-SID-INI1 mutants inhibited SIV intracellular viral protein synthesis by ∼1 log (8- to 10-fold), and particle production was also reduced by a similar ∼1 log (6- to 10-fold) (Fig. 2A and B). Furthermore, the SIV virus particle production efficiency remained the same in the presence of either HA-INI1 or HA-SID-INI1, indicating that the HA-SID-INI1 mutants specifically inhibit HIV-1 but not SIV particle production efficiency (Fig. 2C).

The SID-INI1 mutants harbor double substitution mutations. Therefore, we separated the point mutations borne by SID-INI1-B (G69A and V336E) and tested them individually to determine whether the single mutants also inhibited HIV-1 late events. The intracellular viral protein levels, particle production, and particle production efficiency were inhibited in a manner and degree very similar to the effects of the parent SID-INI1-B clone, indicating that both mutations independently caused the same effect (Fig. 2D to F).

The assembly and particle production efficiency of viral particles are highly dependent on the intracellular concentrations of pr55Gag (40). The HA-SID-INI1 mutants inhibited intracellular Gag levels, which could account for the decrease in assembly and particle production (Fig. 2B). To address the question of whether the effect of HA-SID-INI1 on particle production was in part due to reduced intracellular Gag levels, we carried out a control experiment in which the intracellular and virion-associated Gag levels in the cells expressing SID-INI1 were matched to those of controls expressing HA-INI1 that were transfected with decreasing amounts of viral DNA (Fig. 2G to I). The levels of intracellular, and virion-associated p24 were determined, and the particle production efficiency was calculated as the fractions of p24 present in the culture supernatant and intracellular compartments, expressed as the percentage of the total, as described above (Fig. 2G to I). The results indicated that when the intracellular Gag concentrations of the controls (in the presence of HA-INI1) were matched to those of HA-SID-INI1-expressing cells, the difference between the virus particle production efficiency of the control cells and those that expressed SID-INI1 decreased. The particle production efficiency of the controls was slightly higher than the particle production efficiency observed in the presence of HA-SID-INI1, suggesting that a major effect of SID-INI1 was to reduce the intracellular Gag protein levels and that the effect on particle production efficiency was a minor effect (Fig. 2H and I, indicated by dotted arrows). For example, there was an ∼1-log (∼9-fold) reduction in the virus particle production in the SID-INI1-treated cells compared to the level in the control cells expressing similar Gag levels when 5 μg of viral DNA was transfected. However, the difference between the control and SID-INI1 could be less than 1 log, given the fact that the cells transfected with 5 μg of DNA had a slightly higher steady-state level of intracellular Gag than the SID-INI1-expressing cells. These results indicated that a major effect of SID-INI1-B is to reduce the intracellular Gag levels.

**FIG 3:**
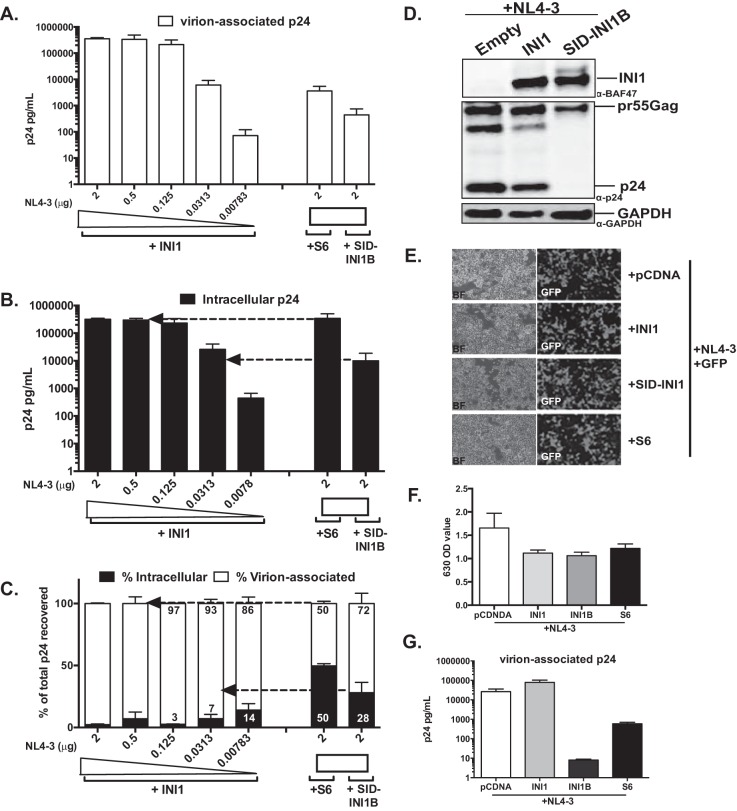
Effects of SID-INI1 on particle production of HIV-1_NL4-3_. (A to C) Quantitation of virion-associated (A) and intracellular (B) p24, and virus particle production efficiency as measured by calculating the percentages of virion-associated and intracellular p24 fractions compared to total p24 (C). (D) Immunoblot analysis to demonstrate the levels of INI1 and SID-INI1-B in transfected cells. The top and middle panels represent two different exposures of the blots using anti-BAF47 antibody, which recognizes both endogenous and transfected HA-INI1 and HA-SID-INI1-B proteins. The results in these panels show similar levels of overexpression of both INI1 and SID-INI1-B compared to the level of GAPDH (bottom). The expression of Gag is detected using anti-p24 antibodies. (E) Photomicrographs illustrating similar transfection efficiencies with empty vector and vectors expressing INI1, SID-INI1, and S6. The DNA was mixed with 1/10 the amount of pEGFP plasmid to allow visualization. (F) Methylene blue uptake assay of cells transfected with empty vector or vector expressing HA-INI1 or HA-SID-INI1-B. The cells were stained with methylene blue, and the OD_630_ was measured from these cells upon lysis. Note the similar levels of decrease in methylene blue uptake in cells transfected with INI1, SID-INI1-B, or S6. (G) Virus production from the cells used for analysis of methylene blue uptake. Supernatants from cells to be treated with methylene blue (as described in the legend to panel F) were collected, and p24 ELISA was performed to determine the amount of virus released from these cells. The arrows point to the control sample with Gag levels comparable to that for the S6- or SID-INI1B-transfected cells.

We next tested the effect of HA-SID-INI1-B on the particle production of full-length molecular clones of HIV-1_NL4-3_. Since HA-SID-INI1-B reduces the intracellular Gag levels, we transfected cells with decreasing amounts of pNL4-3 DNA, along with wild-type INI1, to match the levels of Gag to that observed when HA-SID-INI1-B was transfected. When equal amounts of HA-SID-INI1-B- or HA-INI1-expressing constructs were transfected along with a constant amount of pNL4-3 DNA (2 μg/transfection/well), HA-SID-INI1-B resulted in an ∼3-log reduction in particle production and an ∼1-log reduction in intracellular Gag levels compared to that of the control cells expressing HA-INI1 (Fig. 3A to C). It was interesting to note that even very small amounts of pNL4-3 resulted in significant levels of particle production efficiency (Fig. 3C). When the intracellular Gag levels were matched by transfecting cells with decreasing concentrations of pNL4-3, the particle production efficiency in the cells expressing HA-SID-INI1 was lower than in those expressing HA-INI1 (Fig. 3B and C, indicated by dotted arrows). However, the difference was ∼1 log (∼13-fold) compared to the results for controls with similar intracellular Gag levels. These results again demonstrated that the major effect of HA-SID-INI1-B-mediated inhibition was the reduction in intracellular Gag levels, with an additional minor effect on particle production efficiency. Thus, the SID-INI1 mutant influenced the particle production efficiency of the HIV-1 full-length molecular clone.

As a comparison to HA-SID-INI1-B, we tested the effect of another INI1 mutant, HA-S6 (aa 183 to 294), in the same set of experiments (16). Unlike SID-INI1-B, which is a full-length protein with point mutations, S6 is a truncation mutant harboring the minimal IN-binding domain that when ectopically expressed causes a decrease in the particle production efficiency of 3pV (16). We found that S6 expression did not inhibit the intracellular Gag levels of HIV-1_NL4-3_, but it decreased the particle production by ∼1 to 2 log (range, 20- to 200-fold; mean, 136-fold; Fig. 3A) compared to the level in the matched control, and it also reduced the particle production efficiency (Fig. 3A to C). These observations indicated that, while the HA-SID-INI1 mutant inhibited both intracellular Gag levels and particle production, S6 did not significantly inhibit intracellular Gag levels but did specifically inhibit particle production efficiency.

Western analysis of transfected cells also indicated that similar levels of HA-INI1 and HA-SID-INI1-B proteins were present in transfected cells, and these levels were severalfold greater than the levels of endogenous INI1 protein (Fig. 3D). We measured the viability of cells cotransfected with either HA-INI1 or HA-SID-INI1-B and pNL4-3 DNA by using the methylene blue assay and compared them to the viability of cells cotransfected with the empty vector control and pNL4-3 (Fig. 3F and G). Similar levels of transfection efficiency in these cells were indicated when a GFP-expressing plasmid was added to the mix (1/10th of the total DNA used for transfection) and the expression of GFP monitored. The results indicated that the transfection efficiencies of the controls and test sample were similar (Fig. 3E). Transfection of HA-INI1, HA-SID-INI1-B, or HA-S6 resulted in very similar twofold reductions in cell viability compared to the results for the empty vector control (Fig. 3E and F). However, HA-SID-INI1-B or HA-S6 expression resulted in a 2- to 3-log decrease in particle production compared to the results for the HA-INI1 control (Fig. 3F and G). These results indicate that HA-SID-INI1-mediated inhibition is not due to a decrease in cell viability.

### SID-INI1-B-mediated inhibitory effect is dependent on the presence of IN within the context of Gag-Pol and is independent of PR.

**FIG 4:**
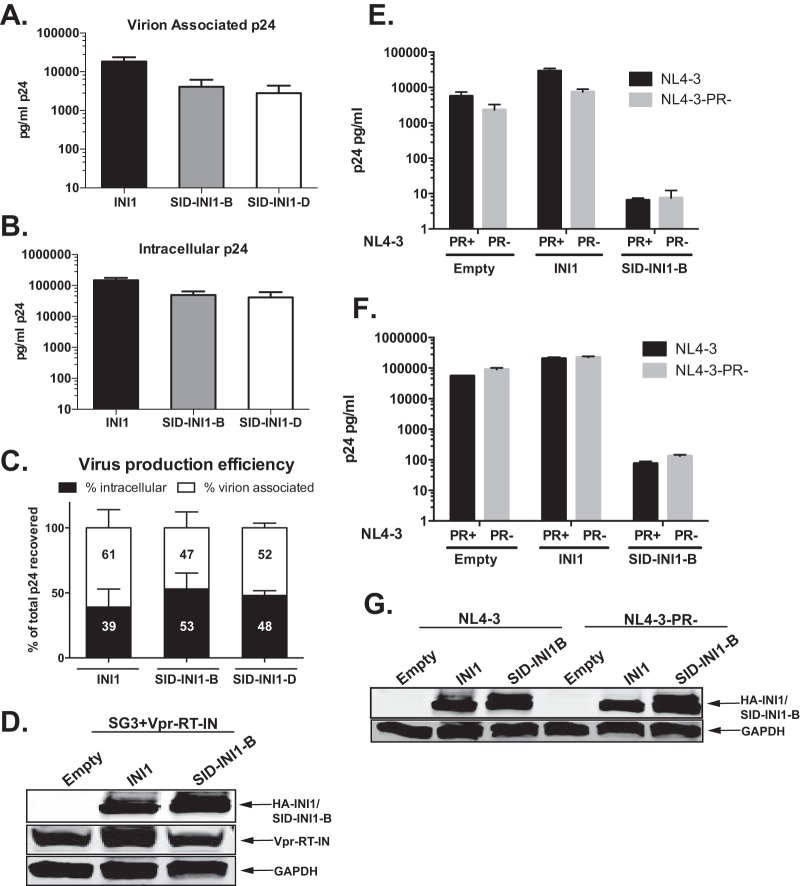
Effects of SID-INI1 mutants on HIV-1 harboring IN deletions or PR mutations. (A to D) Effects of SID-INI1 mutants on SG3-IN+Vpr-RT-IN. (E to G) Effects of SID-INI1 mutants on virus harboring protease mutation (HIV-1_NL4-3_-PR−). (A to C) 293T cells were transfected with SG3-IN+Vpr-RT-IN in the presence and absence of INI1, SID-INI1-B, or SID-INI1-D. Virion-associated p24 in culture supernatants and intracellular p24 in the producer cells were measured by p24 ELISA. Graph represents mean values from 3 independent experiments ± SEM. (D) Western analysis demonstrates the presence of similar levels of Vpr-RT-IN, as compared to the GAPDH level, as well as the presence of INI1 and SID-INI1. (E to F) 293T cells were transfected with pNL4-3 or pNL4-3-PR− along with empty vector or vector expressing INI1 or SID-INI1-B. Virion-associated p24 in culture supernatants and intracellular p24 in the producer cells were measured by p24 ELISA. Graph represents mean values from 3 independent experiments ± SEM. (G) Western analysis to determine the expression of INI1 and SID-INI1 proteins.

Our previous studies indicated that the inhibition mediated by the S6 *trans* dominant-negative mutant required IN to be present in the context of Gag-Pol (16). To determine whether the HA-SID-INI1-mediated effects were also dependent on the presence of IN, we carried out a *trans* complementation assay using SG3-IN virus, which carries a TAA translational stop codon at the beginning of the IN open reading frame and, hence, expresses a truncated Gag-Pol lacking IN. We complemented the lack of IN with Vpr-RT-IN (37). We surmised that if HA-SID-INI1-mediated inhibition is dependent on the presence of IN within the context of Gag-Pol, then the lack of IN would decrease its inhibitory effects. The HA-SID-INI1 mutants decreased the intracellular viral protein levels marginally, by ∼5- to 10-fold, and the viral particle production was decreased similarly, by ∼10-fold, compared to the results for the empty vector control (Fig. 4A and B). Thus, we did not see an additional decrease in particle production in the absence of IN, and there was no significant effect on particle production efficiency compared to that of the control (Fig. 4C). These results indicated that the SID-INI1 mutants did not inhibit particle release when IN was removed from Gag-Pol, suggesting that the SID-INI1-mediated inhibition requires IN to be present within Gag-Pol (Fig. 4A to C). Western analysis confirmed the expression of similar levels of Vpr-RT-IN in the transfected cells when normalized to glyceraldehyde-3-phosphate dehydrogenase (GAPDH) levels, suggesting that when IN is not a part of Gag-Pol, SID-INI1-B does not decrease the intracellular levels of this protein (Fig. 4D).

Previous studies have indicated that certain class II mutations of IN lead to the inhibition of HIV-1 particle production due to inappropriate viral PR activity and that PR inhibitors or inactivating mutations (such as N25A) in the *PR* gene can overcome these inhibitory effects (12). These previous results suggested that mutations in IN may stimulate inappropriate PR activity within the cells and may lead to the inhibition of assembly. Since SID-INI1 mutants retain their ability to bind to IN, these mutants could mimic the effect of IN mutants and could decrease intracellular Gag levels by inducing inappropriate activation of PR. Therefore, we tested the effect of SID-INI1 in the presence of a catalytically inactivating mutation in PR (D25A mutant; PR−) in HIV-1_NL4-3_ that was previously shown to block PR activity (36). Our results indicated that SID-INI1-mediated effects could not be overcome by abrogating PR activity (Fig. 4D and E). The two results described above indicated that the inhibition mediated by SID-INI1 required the presence of IN within Gag-Pol and was not due to the inappropriate activation of PR.

### SID-INI1 mutants do not affect LTR transcription.

**FIG 5:**
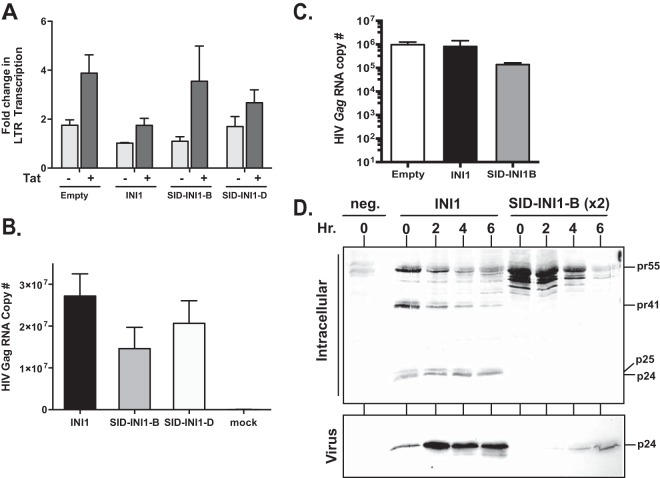
Effects of SID-INI1 mutants on LTR-mediated transcription and viral *gag* RNA and protein levels. (A) Graph illustrating the fold changes in Tat-mediated activation of LTR-Luc in the presence of empty vector, INI1, or SID-INI1-B or -D and in the presence or absence of HIV-1 Tat. (B) Effects of SID-INI1 mutants on the steady-state *gag* RNA levels expressed from 3pV. qRT-PCR was carried out to quantify the HIV-1 *gag* RNA copy numbers of HIV-1 3pV in the presence of INI1 or SID-INI1 mutants. Mock, untransfected control. (C) Effects of SID-INI1 on the steady-state *gag* RNA levels expressed from HIV-1_NL4-3_. qRT-PCR was carried out to quantify the HIV-1 *gag* RNA copy number of HIV-1_NL4-3_ in the presence of empty vector, INI1, or SID-INI1. (D) Pulse-chase analysis to determine the effect of SID-INI1 on HIV-1 Gag stability. Photomicrograph illustrates ^35^S-labeled cellular lysates (top) and viral supernatants (bottom) collected at 0, 2, 4, and 6 h postchase and immunoprecipitated with anti-p24 antibody. Neg., cells were transfected with HA-INI1 only and no HIV-1 (negative control). For the experiments using SID-INI1-expressing cells, twice the amount of protein was loaded in the gel compared to the amount for the control, to be able to detect the low levels.

Since INI1 has multiple roles during HIV-1 replication, we investigated the effects of SID-INI1 mutants on transcriptional and posttranscriptional events that could subsequently impact Gag/Gag-Pol levels and influence assembly and particle release. INI1/hSNF5, as a part of the SWI/SNF complex, regulates HIV-1 LTR transcription. While it inhibits basal-level transcription, it is required for Tat-mediated transactivation (41). Therefore, SID-INI1 mutants could inhibit LTR transcription to inhibit late events. We tested the effect of the SID-INI1 mutant on LTR transcription using an LTR-Luc construct in the presence and absence of Tat in 293T cells. Our results indicated that the SID-INI1 mutants did not interfere with Tat-mediated transactivation of LTR (Fig. 5A). Interestingly, in our experimental setting, HA-INI1 seems to slightly inhibit both basal and Tat-mediated transcription. In contrast, the expression of HA-SID-INI1 did not affect either basal or Tat-mediated transcription.

In addition to testing the effect on LTR transcription, we tested the effect of HA-SID-INI1 on RNA levels by using qRT-PCR. First, we used a 3-plasmid-based vector (3pV) for this purpose, since the viral RNAs encoding Gag and Gag-Pol are expressed from a CMV promoter that is unaffected by INI1 (32). Thus, any effect on *gag* RNA that we would observe by expressing SID-INI1 from this vector was likely be due to its effect on posttranscriptional events. We found that the level of total *gag* RNA from 3pV was slightly reduced (by ∼1.5- to 2-fold) when SID-INI1 was expressed in the cells (Fig. 5B). These results indicated that the HA-SID-INI1 mutants did not affect LTR-mediated transcription and only marginally decreased the total *gag* RNA levels.

We also tested the effects of SID-INI1-B on the steady-state levels of total *gag* RNA produced by HIV-1_NL4-3_. Cells were cotransfected with pNL4-3 in the presence of INI1, SID-INI1-B, or an empty vector. The number of *gag* RNA molecules in each sample was quantitated by normalizing the values using a standard curve. The results of this analysis indicated that the presence of INI1 did not affect the level of *gag* RNA compared to the results for the empty vector control (Fig. 5C). However, the presence of SID-INI1-B decreased the steady-state level of *gag* RNA by 5- to 10-fold compared to the presence of the empty vector or INI1 control (Fig. 5C). These results indicated that while SID-INI1-B did not have an effect on LTR-mediated transcription, its presence decreased *gag* RNA levels to some extent. These results suggest a posttranscriptional effect of SID-INI1 and an additional effect of this mutant on *gag* RNA stability.

### SID-INI1 mutants inhibit posttranslational Gag stability.

To determine whether SID-INI1-mediated effects were manifested during translational or posttranslational events, we carried out a pulse-chase analysis of cells transfected with HIV-1 DNA along with vectors expressing INI1 or SID-INI1-B. Posttransfection, the cells were pulsed with [^35^S]methionine-[^35^S]cysteine for 1 h and chased for various times, and the total intracellular and virion-associated ^35^S-labeled proteins were collected at 0, 2, 4, and 6 h postchase, immunoprecipitated using anti-p24 antibodies, and separated on SDS-PAGE gels (30). The results indicated that in control cells expressing INI1, the Gag precursor pr55, intermediates p41 and p25, and fully processed p24 proteins were present at early time points (Fig. 5D, top). At later time points, the amounts of unprocessed pr55 and intermediates (p41 and p25) decreased in the cells and p24 accumulation increased in the culture supernatants, indicating processing, assembly, and viral particle release (Fig. 5D, top and bottom). In SID-INI1-expressing cells, significant amounts of pr55 Gag were present at 0 h and 2 h postchase (Fig. 5D, top). However, contrary to the results for the control cells, no processed intermediates were seen at either the early or late time points (Fig. 5D, top). Instead, the pr55 proteins degraded rapidly over time and very little p24 accumulated in the virion-associated culture supernatants (Fig. 5D, bottom). These results indicated that a dramatic inhibition of Gag levels was caused by the expression of SID-INI1.

### SID-INI1 mutant inhibits the accumulation of Gag/Gag-Pol at the membrane.

**FIG 6:**
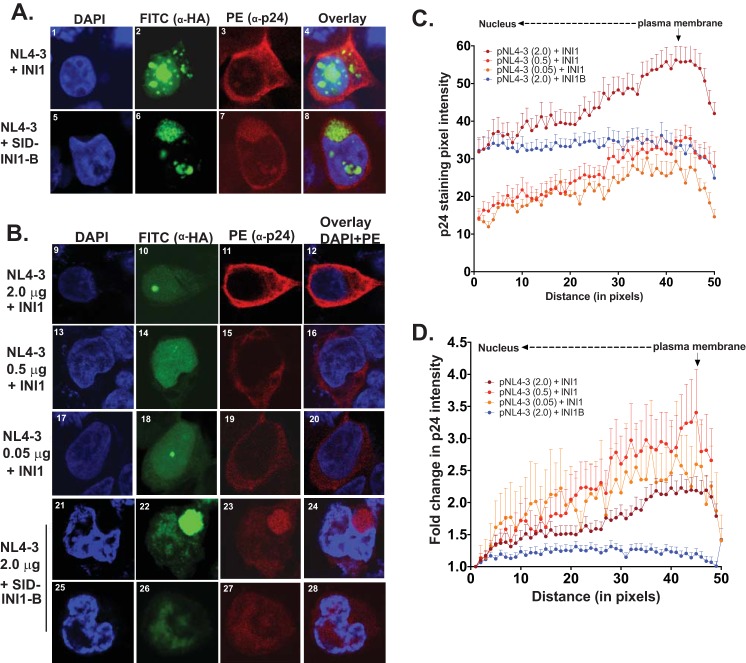
Effect of SID-INI1-B on subcellular localization of Gag/Gag-Pol proteins. (A and B) Effect of SID-INI1 on Gag membrane targeting. Confocal images of cells transfected with equal amounts (2.0 μg each) (A) or decreasing amounts (2.0, 0.5, and 0.05 μg) (B) of pNL4-3 and costained with anti-HA and anti-p24 antibodies. DAPI staining of nuclei, FITC fluorescence indicating staining with anti-HA antibodies, PE fluorescence indicating staining with anti-p24 antibodies, and overlay of the three channels (A) or of DAPI and FITC channels (B) are indicated above the columns. (A) Exposure settings were as follows: DAPI (blue), gain, 814, and offset, −2.5; HA (FITC, INI1/INI1-B, green), gain, 993, and offset, −1.6; p24 (PE, red), gain, 1,250, and offset, −1.7. (B) Exposure settings were as follows: DAPI (blue), gain, 924, and offset, −2.5; HA (FITC, INI1/INI1-B, green), gain, 564, and offset, −0.55; p24 (PE, red), gain, 1,097, and offset, −0.39. (C and D) Graphic representation of the mean fluorescence intensities (±SEM) of p24 across the cytoplasm in cells transfected with INI1 or SID-INI1, with distance represented in pixels. (C) Plot of the mean gray values (±SEM) representing the pixel intensities across the cytoplasm of various control cells transfected with 2.0 (*n* = 34), 0.5 (*n* = 15), or 0.05 (*n* = 14) μg of pNL4-3 and INI1 or with 2.0 μg of pNL4-3 and SID-INI1-B (*n* = 45). (D) As described for panel C but representing the normalized gray values, where the first data point is assigned a value of one and the remaining gray values are expressed as fold changes compared to the first one from the same cell. This allows the comparison of cells with different pixel intensities. The graph represents the mean values (±SEM).

Since reduced levels of Gag are likely to affect subsequent events of assembly, we used confocal microscopy to visualize the Gag localization in control cells and cells transfected with SID-INI1-B. To investigate the effects of reduced Gag levels in the cells transfected with HA-SID-INI1-B on Gag/Gag-Pol localization, we used three different concentrations of pNL4-3 to cotransfect with the HA-INI1 control. The cells from these different transfections were subjected to immunofluorescence analysis using anti-HA (conjugated to FITC) and anti-p24 (with PE-conjugated secondary) antibodies, followed by confocal microscopy imaging and analysis. The cells were also stained with DAPI to detect nuclei. In control cells expressing HA-INI1 and HIV-1_NL4-3_, INI1 localized diffusely in the nucleoplasm, with distinct large and small nuclear speckles, and a small amount of protein was diffusely present in the cytoplasm (Fig. 6A and B, panels 2 and 10). In the majority (>95%) of these cells, p24 accumulated at the cellular periphery, along the plasma membrane, indicating plasma membrane targeting of Gag/Gag-Pol (Fig. 6A and B, panels 3 and 11). In contrast, HA-SID-INI1-B protein accumulated in the nucleus in the less dense region of the chromatin and also in the perinuclear compartments as large patches (in ∼83% of the cells [*n* = 88]; Fig. 6B, panels 22 and 26). In other cells, it accumulated in the nucleus and localized diffusely in the cytoplasm (in ∼17% of the cells; Fig. 6A, panel 6).

In >90% of the control cells, Gag accumulated sharply at the membrane, whereas in 100% of SID-INI1-B-transfected cells, the localization of Gag was diffuse, and it often colocalized with SID-INI1-B (Fig. 6A, panels 3 and 7). Since the concentration of Gag affects membrane accumulation, we tested the effect of decreasing the concentration of viral DNA by transfecting 2.0, 0.5, and 0.05 μg each of HIV-1 DNA, along with HA-INI1. The control cells with decreasing Gag levels, as well as cells transfected with HIV-1 (2.0 μg) and SID-INI1-B, were imaged using identical settings (see the legend to Fig. 6). To determine the distribution of Gag in these control cells (*n* = 34) and in those expressing SID-INI1 (*n* = 45), the images were analyzed by quantitating the pixel intensity (gray value) of Gag protein staining obtained using anti-p24 antibodies, as described in detail in earlier reports (30, 42). The gray value is the intensity of the brightness in any given pixel. Black is the absence of signal (a gray value of 0), while white is the maximum (saturation, a gray value of 256) brightness of a pixel. An 8-bit-thick line was drawn across the cytoplasm from the membrane, the gray values were determined across this line for a distance of 50 pixels, and the means of these values were plotted across the distance in pixels (Fig. 6C). The distribution of gray value intensities in control cells expressing various Gag levels indicated that there was a trend of accumulation of Gag at the plasma membrane, even when the intensities were very low (as in cells transfected with 0.5 and 0.05 μg DNA) (Fig. 6C). This trend was clear when the gray values were expressed as the fold changes in the intensities across the distance in pixels within each cell and the means of these values were plotted (Fig. 6D). Interestingly, the distribution of Gag in cells transfected with SID-INI1-B was distinctly different from that of the controls and exhibited a diffuse pattern across the cytoplasm (Fig. 6C and D). These results suggested that SID-INI1-B expression affects the Gag/Gag-Pol cytoplasmic localization and accumulation at the plasma membrane.

### Knocking down INI1 reduced Gag/Gag-Pol levels and particle production.

The results described above demonstrated that the HA-SID-INI1 mutant reduced Gag levels and inhibited its accumulation at the membrane, but these experiments did not elucidate whether endogenous wild-type INI1 was required for these events. To address the requirement of wild-type INI1 for late events, we tested the effect of knocking down endogenous *INI1* in 293T cells. It is known that complete knock down of INI1 is toxic to cells, and hence, we could achieve only a partial knockdown in order to prevent cell death. Furthermore, the cells were monitored at every stage of the transfection and tested for viability by using the methylene blue assay. Both Western analysis and single-cell immunofluorescence analysis using anti-INI1 antibody were employed to monitor the level of INI1 knockdown, as described below (43).

**FIG 7:**
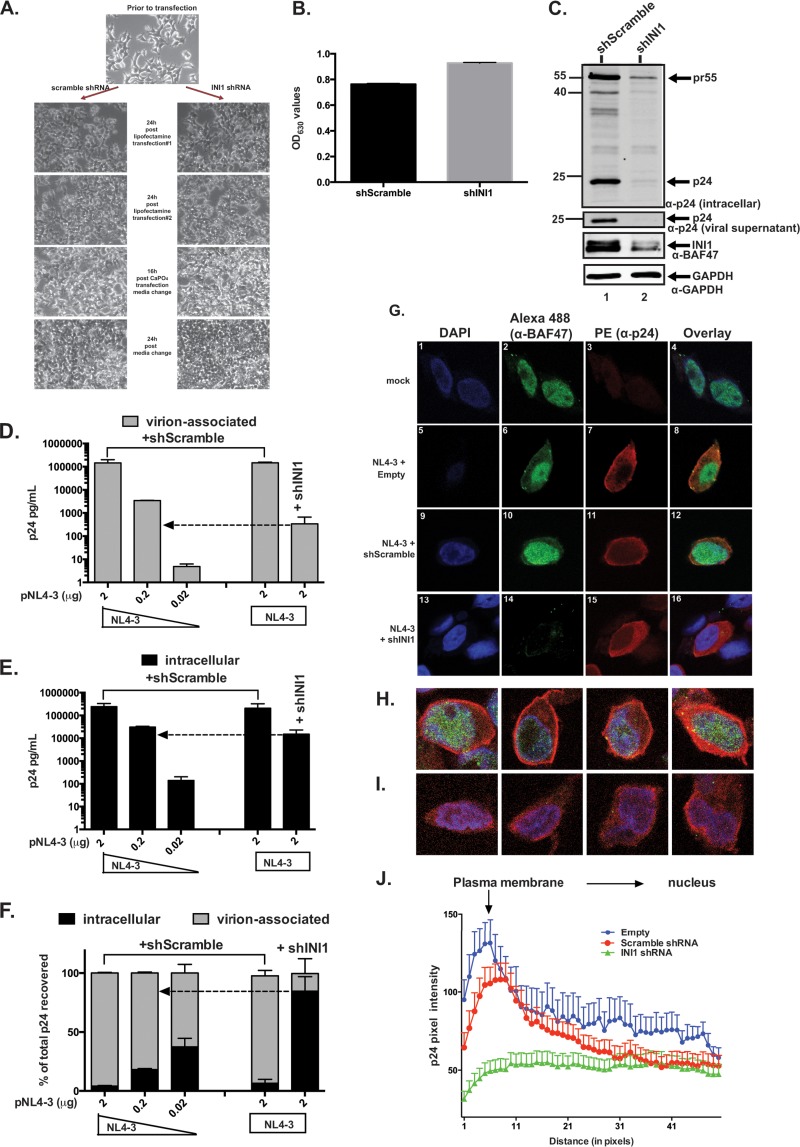
Knocking down INI1 in 293T cells leads to inhibition of Gag/Gag-Pol stability and particle production. (A) Photomicrographs of cells at various stages of transfection during shRNA analysis. All images were taken at ×20 magnification. (B) Methylene blue uptake assay. OD_630_ values, representing the methylene blue uptake for cells transfected with shScramble and sh-INI1 along with pNL4-3, are indicated. (C) Immunoblot analysis to determine the knockdown of INI1. Total proteins from cells transfected with pNL4-3 along with either shScramble or shINI1 were subjected to immunoblot analysis using anti-p24 antibody to detect Gag proteins, anti-BAF47 antibody to detect INI1, and anti-GAPDH antibody to detect GAPDH as an internal control. (D to F) Effects of INI1 shRNA on virus particle production in 293T cells. Quantitation of virion-associated p24 (D) and intracellular p24 (E) and virus production efficiency (F), as described in the legend to Fig. 2, in the presence of either shScramble (control) or shINI1. (G) Confocal images of cells transfected with either shScramble or shINI1, cotransfected with HIV-1_NL4-3_, and costained with anti-BAF47 (INI1) and anti-p24 antibodies. DAPI staining of nuclei, Alexa Fluor 488 fluorescence indicating staining with anti-BAF47 (INI1) antibody, PE fluorescence indicating staining with anti-p24 antibody, and overlay of the three are indicated above the columns. The gain and offset settings for these images were as follows: DAPI (blue), gain, 984, and offset, −2.3; Alexa Fluor 488 (green), gain, 1,244, and offset, −2.7; PE (red), gain, 1,250, and offset, −1.18. (H and I) Additional images (overlay of all three channels) of cells transfected with shScramble (H) and shINI1 (I) along with pNL4-3. (J) Graphic representation of the mean fluorescence intensities (±SEM) of p24 across the cytoplasm in cells transfected with empty vector (*n* = 7), shINI1 (*n* = 47), or shScramble (*n* = 34), with distances represented in pixels. The arrows point to the control sample with Gag levels comparable to that for the shINI1-transfected cells.

To determine the effect of INI1 knockdown on HIV-1 late events, cells were transfected twice with shRNA-expressing plasmids, followed by a third round of transfection with shRNAs and pNL4-3 plasmids. Our analysis indicated that the viability of transiently transfected cells was unaffected by shRNA against INI1 (shINI1) during the course of these experiments and was identical to that of scrambled control shRNA-transfected cells (Fig. 7A). A methylene blue uptake assay indicated that the viability of cells transfected with scrambled shRNA or shINI1 was similar (Fig. 7B). Immunoblot analysis indicated that INI1 knockdown led to a decrease of ∼80% in the protein level (Fig. 7C). When similar amounts of pNL4-3 DNA (2 μg) were used in control- and shINI1-transfected cells, the knockdown of INI1 led to a reduction of intracellular Gag levels by approximately 10-fold compared to the results for scrambled shRNA (shScramble) (Fig. 7E, leftmost two bars). In addition, there was a 2- to 3-log decrease in virion-associated p24 in cells transfected with shINI1 (Fig. 7D, leftmost two bars). As a consequence, there was an additional decrease in particle production efficiency in cells transfected with shINI1 (Fig. 7F, leftmost two bars). To determine whether INI1 knockdown directly influenced particle release, the intracellular Gag levels in control cells transfected with shScramble were matched to the levels in cells transfected with shINI1 by using decreasing concentrations of pNL4-3 DNA. The results indicated that even when the intracellular Gag levels were matched to the levels of shScramble- and shINI1-transfected cells, the particle production efficiency in cells transfected with shINI1 was significantly reduced compared to that of the scrambled-shRNA-transfected cells (Fig. 7E and F, indicated by dotted arrows).

The results of confocal microscopy indicated that in empty vector and scrambled-shRNA-transfected cells, Gag accumulated at the cell membrane in ∼90% of the cells (Fig. 7G, panels 7 and 11, and H). In these cells, the majority of INI1 was present in the nucleus, but some INI1 was also present in the cytoplasm (Fig. 7G, panels 6 and 10). In contrast, in cells transfected with shINI1, no INI1 was detected, and in these cells, the level of Gag was reduced and it was localized diffusely in the cytoplasm and did not accumulate at the membrane (Fig. 7G, panels 14 and 15, and I). To quantitate the distribution of Gag in the absence of INI1, the fluorescence intensity of p24 staining was quantified across the cytoplasm from the plasma membrane to the nucleus by plotting the gray value as described above and in the legend to Fig. 6 and as reported previously (30, 42). In control cells transfected with shScramble (*n* = 34), the peak of Gag accumulation was found at the plasma membrane and its amounts gradually decreased toward the nucleus (Fig. 7J). In contrast, in cells expressing shINI1 (*n* = 47), the level of Gag was low, no peak intensity of p24 staining was found, and there was an even distribution across the cytoplasm, consistent with the lack of Gag membrane accumulation (Fig. 7F). Thus, RNA interference analysis indicated that down-modulation of *INI1* expression resulted in an impairment of Gag levels and an additional decrease in particle production, suggesting that INI1 is required for both of these processes.

## DISCUSSION

The goal of our studies was to delineate the role of INI1 in HIV-1 replication by understanding the effects of various INI1 mutants and shRNA knockdown of INI1. In previous reports, we demonstrated that an ectopically expressed deletion mutant of INI1, termed S6, dominant-negatively inhibits HIV-1 particle production and Gag/Gag-Pol trafficking (30). However, the question regarding the role of endogenous INI1 in assembly events remained unanswered. In the work presented in this report, we tested the effects of an additional set of INI1 missense mutations (termed SID-INI1 mutants) that were partially or fully defective for interaction with SAP18 while retaining their ability to interact with IN. We also tested the effect of knocking down INI1 on HIV-1 assembly and particle production. Our results indicate that SID-INI1 clone B9 (SID-INI1-B) inhibits multiple posttranscriptional events of HIV-1 replication. Its major effect is to reduce the steady-state levels of intracellular Gag protein. In addition, SID-INI1-B reduces viral RNA levels but not LTR-mediated transcription, and it reduces the particle production efficiency. Knocking down INI1 also results in reduced Gag protein levels and decreased particle production. These effects of the expression of SID-INI1-B and of knocking down INI1 on HIV-1 late events are due not to a toxic effect on the transfected cells but, rather, to an effect on the steady-state levels of viral RNA and proteins.

INI1 is a component of the SWI/SNF complex and has been shown to regulate LTR-mediated transcription (27). Our studies indicate that mutants of INI1 impaired for binding to SAP18 affect posttranscriptional events, which is unprecedented. The inhibitory effect of SID-INI1 mutants on assembly and particle production are largely due to their effects on Gag RNA and protein levels. While the SID-INI1 mutant does not decrease LTR-mediated transcription, qRT-PCR analysis indicates that RNA levels are affected, suggesting a posttranscriptional effect. Furthermore, pulse-chase analysis indicates that in the presence of SID-INI1, Gag protein that is translated is rapidly degraded, suggesting that the effects are posttranslational. A minor effect of SID-INI1 is also to reduce the particle production efficiency. Since assembly is a cooperative process and is dependent on intracellular Gag levels, the SID-INI1-mediated reduction in particle production efficiency could be a reflection of reduced intracellular Gag levels. Interestingly, the S6 mutant, which does not drastically inhibit intracellular Gag levels, nevertheless inhibits particle release, suggesting a specific effect on particle production efficiency. Our previous results indicate that S6 inhibits the accumulation of Gag at the membrane (30). In this regard, the two mutants show differential effects—S6 specifically decreases particle production efficiency, and SID-INI1 mutants drastically reduce intracellular Gag levels, which leads to an additional reduction in particle release.

The SID-INI1-mediated inhibitory effects are dependent on the presence of IN within the context of Gag-Pol. This result correlates with our previous study of S6-mediated inhibition (16). The degree of SID-INI1-mediated inhibition is reduced when SIV-based vectors are used, consistent with the finding that INI1 specifically binds to HIV-1 but not to SIV IN (17). It is possible that the selective binding of the mutant SID-INI1-B to HIV-1 IN (but not to SIV IN) mediates this inhibition. In addition, SID-INI1 did not dramatically inhibit HIV-1 particle production in a *trans* complementation assay using an IN deletion mutant virus complemented with Vpr-RT-IN (SG3-IN+Vpr-RT-IN). In these experiments, the intracellular levels of Gag were reduced approximately threefold by SID-INI1, and particle production was inhibited approximately fivefold. Hence, there was no dramatic decrease in particle production efficiency caused by the SID-INI1 mutant when SG3-IN+Vpr-RT-IN was used. These results suggest that the SID-INI1-mediated reduction in Gag levels and inhibition of particle release are both dependent on the presence of IN within the context of Gag-Pol. An alternative possibility is that IN may not be required, but since mutant Gag-Pol that lacks IN may not be folded properly, the SID-INI1 mutant cannot perform its inhibitory function. While this argument indicates that the presence of intact and properly folded Gag-Pol may be required for SID-INI1-mediated inhibition, it is not supported by the fact that SIV Gag-Pol, which is likely to be folded properly, is not inhibited by SID-INI1-B, thus suggesting that the presence of IN is required for SID-INI1-mediated inhibition. However, at this point, it is unclear why there was a 1-log reduction in the intracellular levels of SIV Gag, which did not lead to further inhibition of particle production. One possibility is that SID-INI1-B inhibits SIV Gag RNA levels (and, hence, the Gag protein level) but does not inhibit particle production efficiency, as the later requires binding to IN.

Our results, for the first time, indicate that endogenous INI1 is required in maintaining Gag levels and for particle production. shRNA-mediated inhibition of endogenous INI1 in 293T cells results in reduced Gag/Gag-Pol levels. Furthermore, there is an additional decrease in particle production efficiency. This was confirmed by matching the intracellular Gag levels in control cells expressing scrambled shRNA to the levels in the cells expressing shINI1. These results suggest that functional INI1 is required to maintain Gag levels and particle production.

The two classes of the SWI/SNF complex, the BAF and PBAF complexes, have been shown to influence both basal and Tat-mediated transcription of HIV-1 LTR (27, 44). While the BAF complex inhibits basal-level transcription, the PBAF complex is required for Tat-mediated transactivation. INI1 is a component of both the BAF and PBAF complexes. It is interesting to note that in our experimental system, SID-INI1 does not significantly affect either the Tat-dependent or Tat-independent transcription of the reporter LTR-Luc. Compared to cells expressing INI1, SID-INI1 expressing cells have increased levels of LTR transcription. The possible reasons are as follows: (i) SAP18 is a component of the HDAC1 repressor complex and the lack of interaction between INI1 and SAP18 may not decrease transcription from LTR, and/or (ii) SID-INI1 is not localized in the same compartment as the LTR. SID-INI1 is localized in the cytoplasm and nucleus as large patches and speckles. However, SID-INI1 affects *gag* RNA levels, as indicated by the results of qRT-PCR. When *gag* RNA is expressed from a CMV promoter in the 3pV, SID-INI1 mutants marginally decrease the RNA levels, by two- to threefold. When *gag* RNA is expressed by LTR-mediated transcription of full-length virus (pNL4-3), the RNA levels are decreased by 3- to 10-fold. Thus, it appears that while SID-INI1 does not inhibit LTR transcription, it decreases *gag* RNA levels by 1- to 10-fold, depending on the promoter, and additionally decreases Gag protein levels by 10- to 100-fold and particle release by an additional 5- to 10-fold. These results indicate that the inhibitory effects of SID-INI1 are not due to the decrease in LTR transcription but are more likely due to a cumulative effect on posttranscriptional and posttranslational events. Future experiments will shed light on the mechanism of these newly observed effects.

While INI1/hSNF5 and its role in transcription as a component of the SWI/SNF complex are well established, emerging evidence suggests that the components of the SWI/SNF complex are also involved in posttranscriptional events, such as splicing (45). The SWI/SNF complex and INI1/hSNF5 are associated with non-SWI/SNF proteins, as well with multiple cellular substructures, such as nuclear lamin, DNA replication origins, kinetochores, centrosomes, microtubules, and PML bodies, suggesting multiple cellular functions for these chromatin-remodeling proteins (46). Our finding that INI1 mutants affect multiple late events cannot be explained by the role of endogenous INI1 in viral transcription alone. While the effect on transcription perhaps is a part of the inhibition when viral proteins are expressed from the LTR, the data from experiments using mutants and shRNAs collectively indicate multiple posttranscriptional effects.

How does INI1 and its interaction with SAP18 influence HIV-1 late events? Based on our results and on the ability of INI1 to mediate multiple protein-protein interactions, we propose that INI1 may bridge the interactions between Gag/Gag-Pol and cellular proteins like SAP18 and associated complexes via binding to HIV-1 IN. INI1 may function as an adapter molecule that promotes the protein interactions required for these events in viral replication. We hypothesize that these SAP18-associated proteins/complexes are involved in maintaining the stability of *gag* RNA and Gag protein and also influence Gag/Gag-Pol accumulation at the membrane. Efforts are under way in our laboratory to identify INI1-SAP18-associated complexes that may play a role during HIV-1 posttranscriptional late events. The identification of these complexes could provide novel insight into host-virus interactions that influence Gag/Gag-Pol stability, trafficking, and particle release.

The transcription and posttranscriptional events of HIV-1 replication are not currently targets of antiretroviral drug discovery efforts. We suggest that our results provide insights that could be employed to develop novel strategies to inhibit these posttranscriptional events in HIV-1 replication. Since interfering with INI1 influences Gag stability and trafficking, we propose that low-molecular-weight inhibitors that interfere with the INI1-IN interaction or the INI1-SAP18 interaction could possibly inhibit HIV-1 replication by reducing Gag/Gag-Pol stability and particle release.
